# Tools to Enhance Resident and Family Engagement in Assisted Living Safety: A Scoping Review.

**DOI:** 10.1093/geroni/igab046.1332

**Published:** 2021-12-17

**Authors:** Thi Vu, Jennifer Leeman, Marianne Baernholdt, Christine Kistler, Elizabeth Moreton, Terri Ottosen, Pam Dardess, Anna Beeber

**Affiliations:** 1 Yale University, New Haven, Connecticut, United States; 2 University of North Carolina at Chapel Hill School of Nursing, Chapel Hill, North Carolina, United States; 3 University of North Carolina at Chapel Hill Division of Geriatric Medicine and Dept. of Family medicine, Chapel Hill, North Carolina, United States; 4 University of North Carolina at Chapel Hill Health Sciences Library, Chapel Hill, North Carolina, United States; 5 Institute for Patient- and Family-Centered Care, McLean, Virginia, United States; 6 University of North Carolina at Chapel Hill, UNC Chapel Hill, North Carolina, United States

## Abstract

This presentation reports the results of a scoping review which identified and evaluated existing engagement strategies, tools, and interventions for their fit with assisted living (AL). Using the PRISMA criteria, we evaluated 54 empirical studies in assisted living/residential care or nursing homes (NH) for how they engaged families and residents, promoted person-centered and/or safety in AL/NH care, and assessed relevant outcomes (safety, experience, service use, satisfaction with care, health behaviors, and quality of life). The strategies, tools, and interventions aimed to improve residents’ activities of daily living, function, and quality of life. Studies also targeted staff and family caregivers to increase knowledge, improve relationships, and decrease caregiving stress. Overall, the studies reported statistically significant changes in resident quality of life, agitation, antipsychotic use, staff knowledge and job satisfaction. Results from this systematic review will inform the development of a testable toolkit to increase engagement and improve safety in AL.

